# Theoretical study on the structure, spectroscopic, and current–voltage behavior of 11-*Cis* and *Trans* retinal isomers in rhodopsin

**DOI:** 10.1038/s41598-024-63249-8

**Published:** 2024-05-30

**Authors:** Amin Hamedian, Mohammad Vakili, Silvia A. Brandán, Mahmood Akbari, Ayoub Kanaani, Vahidreza Darugar

**Affiliations:** 1 Department of Chemistry, Faculty of Science, Ferdowsi University of Mashhad, Mashhad, 91775-1436 Iran; 2 Cátedra de Química General, Instituto de Química, Inorgánica Facultad de Bioquímica, Química y Farmacia, Universidad Nacional de Tucumán, Ayacucho 471, 4000 Tucumán, Argentina; 3 UNESCO‑UNISA-ITL Africa Chair in Nanoscience and Nanotechnology (U2ACN2), College of Graduate Studies, University of South Africa (UNISA), Pretoria, South Africa; 4 School of Chemistry, Damghan University, Damghan, 36716-41167 Iran

**Keywords:** Rhodopsin, Molecular switch, NEGF-DFT, 11-*Cis* retinal, *Trans* retinal, Physical chemistry, Theoretical chemistry

## Abstract

In this study, the electronic transport properties of 11-*Cis* and *Trans* retinal, components of rhodopsin, were investigated as optical molecular switches using the nonequilibrium Green’s function (NEGF) formalism combined with first-principles density functional theory (DFT). These isomers, which can be reversibly converted into each other, were examined in detail. The structural and spectroscopic properties, including infrared (IR), Raman, nuclear magnetic resonance (NMR), and ultraviolet (UV) spectra, were analyzed using the hybrid B3LYP/6–311 +  + G** level of theory. Complete vibrational assignments were performed for both forms utilizing the scaled quantum mechanical force field (SQMFF) methodology. To evaluate the conductivity of these molecules, we utilized current–voltage (I-V) characteristics, transmission spectra, molecular projected self-consistent Hamiltonian (MPSH), HOMO–LUMO gap, and second-order interaction energies (E^2^). The trendline extrapolation of the current–voltage plots confirmed our findings. We investigated the effect of different electrodes (Ag, Au, Pt) and various connection sites (hollow, top, bridge) on conductivity. The Ag electrode with the hollow site exhibited the highest efficiency. Our results indicate that the *Cis* form has higher conductivity than the *Trans* form.

## Introduction

One of the topics that has attracted significant attention in the last decade is the use of molecules in the production of circuits and electronic components that have small dimensions and bulk scales^1,2^. Recently, some physical properties used in molecular electronics include I–V characteristics^3^, negative differential resistance (NDR)^4^, memory effects^5^, solar cells^6,7^, Polymer Light Emitting Diodes (PLEDs)^8^, and switches^9–13^. Among these properties, the molecular switch is particularly important due to its prominent role in storing and transmitting information^14^.

The basic principle in molecular switching is that there must be two or more stable states that can reversibly convert into each other. In this conversion, the form with higher conductivity and lower resistance is known as “on*”* state, and the form that has lower conductivity and higher resistance is known as the “off “state^15^. This conversion can be included by various factors such as electric field^16^, environmental chemicals^17^, solvents^18,19^, ambient temperature^20^, or light^15,21^. Due to their fast response time, light-driven molecular switches have been the focus of various research efforts^22^. Molecules typically used for single-molecule electronics must have characteristics akin to traditional electronic components (such as wires, transistors, rectifiers, and switches). The connections between molecules and electrodes must be capable of attaching to bulk-scale electrodes, usually made of gold, silver, platinum, and other surfaces (electrodes). The conductivity of a molecule changes according to surrounding environmental conditions such as pH, temperature, pressure, measurement properties of the device, electrode surface structure, and electrode geometry at the molecular scale^23,24^. In this regard, various research studies have investigated the transport properties of single molecules to evaluate their application in molecular electronics as molecular switches^25^. Azobenzene and diarylethene-based photochromic switches have been studied extensively^26,27^. In this project, we investigated the electron transport properties of 11-*Cis* retinal and *Trans* retinal, components of rhodopsin, which are biomolecules.

**Figure 1:**
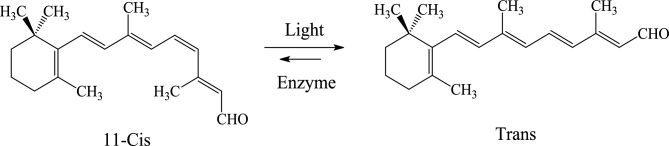
Chemical structures of 11-*Cis* and *Trans* retinal and switching between them.

Rhodopsin is a light-sensitive pigment in the rod photoreceptor cells of the eyes of most vertebrates, playing a crucial role in low-light vision. Rhodopsin is composed of two parts: 11-*Cis*-retinal and a protein part called opsin, covalently linked through a Schiff base bond^28^. Retinal is an aldehyde derivatives of vitamin A. 11-*Cis* retinal is the light-sensitive part of rhodopsin, which is isomerized to the all-trans (*Trans*) retinal form upon receiving visible light, activating rhodopsin and initiating the light detection process^29^. Figure 1 shows the isomerization between the 11-*Cis* and *Trans* forms.

*Trans*-retinal is released by activated rhodopsin, allowing its conversion back to the original 11-*Cis* retinal state. After the primary 11-*Cis* retinal is regenerated, it recombines with opsin, continuing the visual cycle^30,31^. The high sensitivity of 11-*Cis* retinal to light highlights the primary use of rod photoreceptor cells in the eye. Given that these molecules are bio-based and are converted to each other through light, we investigate them as potential compounds for use in molecular electronics as molecular switches. The methodology used in this work has been applied successfully in various other studies^12,13^, and we have customized it by changing conditions such as electrodes and connection sites. Additionally, investigating the structure of rhodopsin can play a crucial role in understanding low-light vision diseases and aiding in their treatment. The spectroscopies results can be used to characterize the structure of molecules, and exploring the structure can help us better understand electron transport properties^32,33^ Since the structure of rhodopsin and the differences between 11-*Cis* retinal and *Trans* retinal are crucial for vision, few experimental works have identified the structure of rhodopsin^34,35^. Therefore, discussing the structure and spectroscopic properties of both rhodopsin isomers theoretically and experimentally is vital for understanding their characteristics and electron transfer behavior.

In this study, we used the non-equilibrium Green's function (NEGF) formalism along with density functional theory (DFT) to investigate the molecular conductivity properties of the target compounds with different electrodes (Ag, Au, and Pt) and three types of adsorptions sites (hollow, bridge, and top), as shown in Fig. S1 (see supplementary materials). The non-equilibrium Green’s function (NEGF) formalism provides a sound conceptual basis for developing atomic-level quantum mechanical simulators needed for future nanoscale devices^36^. In addition, the structural differences and theoretical spectroscopic properties (UV, IR, Raman, and NMR) of both 11- *Cis* and *Trans* forms were also investigated and compared with experimental results to understand the differences and behaviors of both isomers.

## Model and computational methods

According to Zhu et al. fifteen different forms were considered for the *Cis* isomer and one for the *Trans* isomer of retinal^35^. The relative energies and molecular geometries of these forms were optimized using Gaussian 09, Revision D. 01 software^37^ at the B3LYP/6-311 +  + G(d,p) level of theory^38,39^. This level of theory was selected as it has been proven effective in various studies on molecular structures and spectroscopies^40^. The second-order interaction energies (E^2^)^41^ were calculated using NBO 5.0 program^42^, while the vibrational assignments of both isomers were performed using the scaled quantum mechanical force field (SQMFF) methodology, scaling factors, and the Molvib program^43–45^. *GaussView* 5.0 software was used to generate the mapped MEP surfaces^46^. The excitation transitions for titled isomers were calculated using time-dependent density functional theory (TD-DFT)^47^. The ^1^H and ^13^C chemical shifts were calculated using the GIAO (Gauge-Including Atomic Orbital) method^48,49^. The Raman, UV, and NMR spectra were predicted at the same level of theory. Bader’s theory of atoms in molecules was used to study the stabilities of both forms^50^.

**Figure 2:**
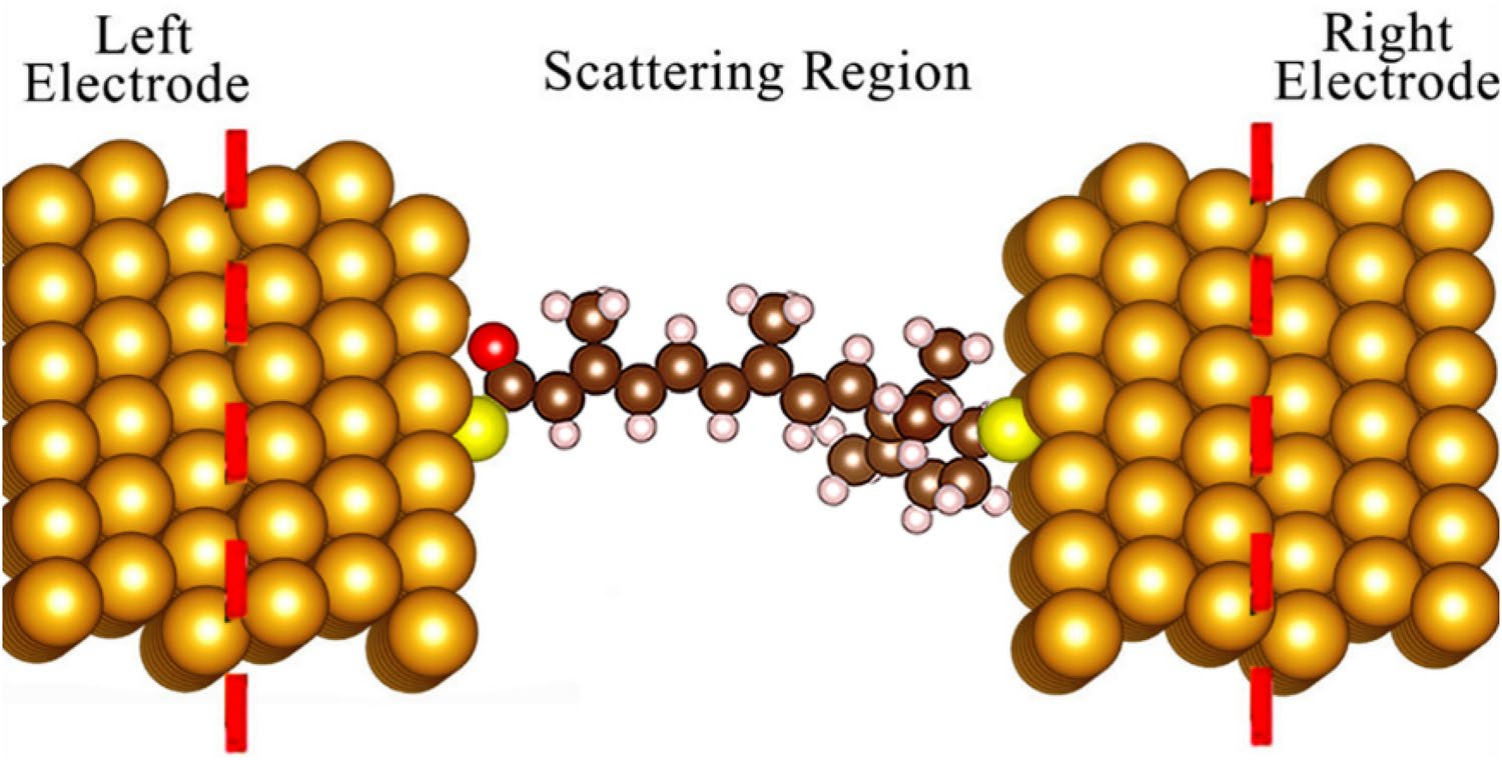
A schematic molecular junction of the Au electrode was used in our calculations (the title molecule attached to the electrode).

The results showed that hydrogen atoms are not attracted to the surface of metal electrodes^51^. Therefore, we replaced the two hydrogen atoms on both sides of the target molecules with SH groups. The target molecules were sandwiched between two parallel Ag, Au, and Pt electrodes with the Y(111) surface^52^ (see Fig. 2). To avoid additional interactions with other molecules, we used surfaces with (6 × 6) periodic boundary conditions in our calculations^53^. In this research, we focused only on the central scattering zone, considering only the atoms in the scattering area within a force convergence criterion of 0.02 eV/Å.

The Perdew–Burke–Ernzerhof (PBE) exchange–correlation functional was applied for the generalized gradient approximation generated (GGA)^54^. We used single-ξ plus polarization and double-ξ plus single polarization (DZP) for the metallic atoms and other elements, respectively. In different switching studies, this level was utilized^9–13^. To increase efficiency in calculations, we used a 3 × 3 × 100 k-grid and set the mesh cutoff to 150 RY^55^. The I-V characteristics and transmission spectra were calculated from 0.0 to 3.0 V at 300 K. The current passing through the device was calculated using the Landauer- Büttiker formula^56^ in the TranSIESTA package^57^:

1 $$I = \frac{2e}{h}\smallint T\left( {E,V} \right)[f\left( {E - \mu_{L} } \right) - f\left( {E - \mu_{R} } \right)]dE,$$

where *e* is the electron charge, ℎ is Planck’s constant, and *T*(*E*, *V*) is the transmission function at energy *E* under bias voltage *V*. (*E* − ${\mu }_{L}$) and *f*(*E* − ${\mu }_{R}$) are the Fermi–Dirac distribution functions corresponding to the electrochemical potential of the left $({\mu }_{L})$ and right ${(\mu }_{R})$ electrodes, respectively.

## Results and discussion

### Isomerism stability and relative energies

In this work, 15 *Cis* and one *Trans* isomers were optimized based on the study reported by Zhu et al.^35^ using Gaussian 09 software at the B3LYP/6-311 +  + G(d,p) level of theory. Their stabilities were compared in the gas phase and water. Table 1 shows the calculated total and ZPVE-corrected energies, dipole moments, and volumes of the most stable forms of 11-*Cis* and *Trans* retinal in the gas phase and water using the B3LYP/6–311 +  + G** level of theory. Polarizabilities (α) and energy differences (ΔE and ΔE _ZPVE_) values between both isomers are also included.

**Table 1:** Calculated total and ZPVE-corrected energies (E), dipole moments (µ), and volumes (V) of 11-*Cis* and *Trans* retinal in the gas phase and aqueous solution by using B3LYP/6–311 +  + G** level of theory.

B3LYP/6–311 + + G** method							
Species	E (Hartrees)	E_ZPVE_ (Hartrees)	µ (D)	V (Å^3^)	α (a.u.)	ΔE (kJ/mol)	ΔE_ZPVE_ (kJ/mol)
Gas phase							
*Cis*	− 854.38495	− 853.95702	7.36	350.3	338.513	19.44	22.03
* Trans*	− 854.39236	− 853.96542	6.08	359.2	349.954	0.0	0.0
Aqueous solution							
* Cis*	− 854.39703	− 853.96968	10.87	360.3	502.659	13.28	15.46
* Trans*	− 854.40209	− 853.97557	9.04	357.8	521.333	0.0	0.0

Polarizabilities (α) and energy differences (ΔE) values are also included.

**Figure 3:**
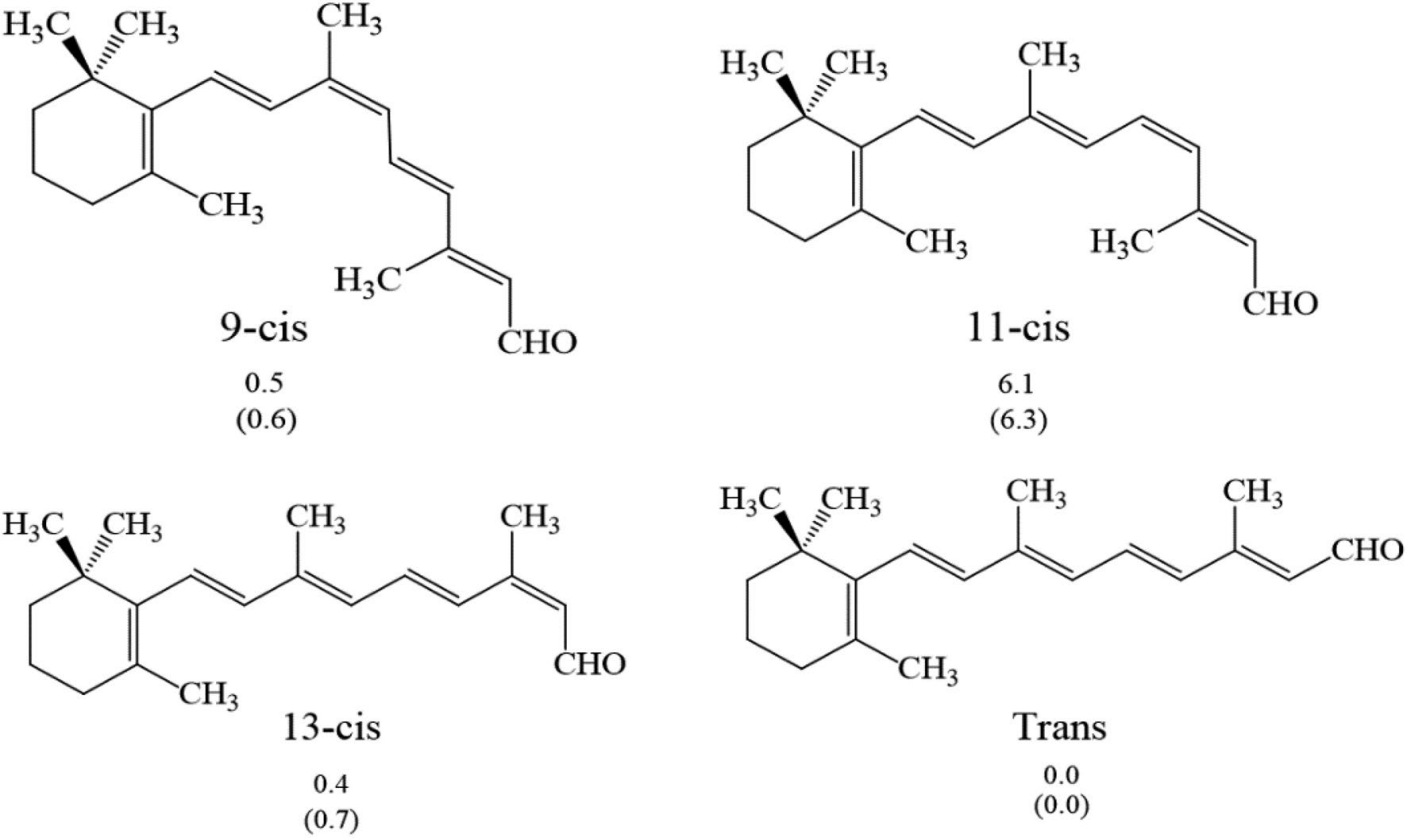
Structures and relative energies (in kcal/mol) in the gas phase and water of the 9- *Cis*, 11- *Cis*, 13-*Cis,* and *Trans* retinal. The values in parentheses are the relative energies in water.

Table 1 shows that the *Trans* form is the most stable in both the gas phase and the aqueous solution. Analyzing the *Cis* isomers, 13- *Cis* retinal in the gas phase and 9- *Cis* retinal in water are the most stable, as shown in Figs. 3 and S2 (see supplementary materials).

**Figure 4:**
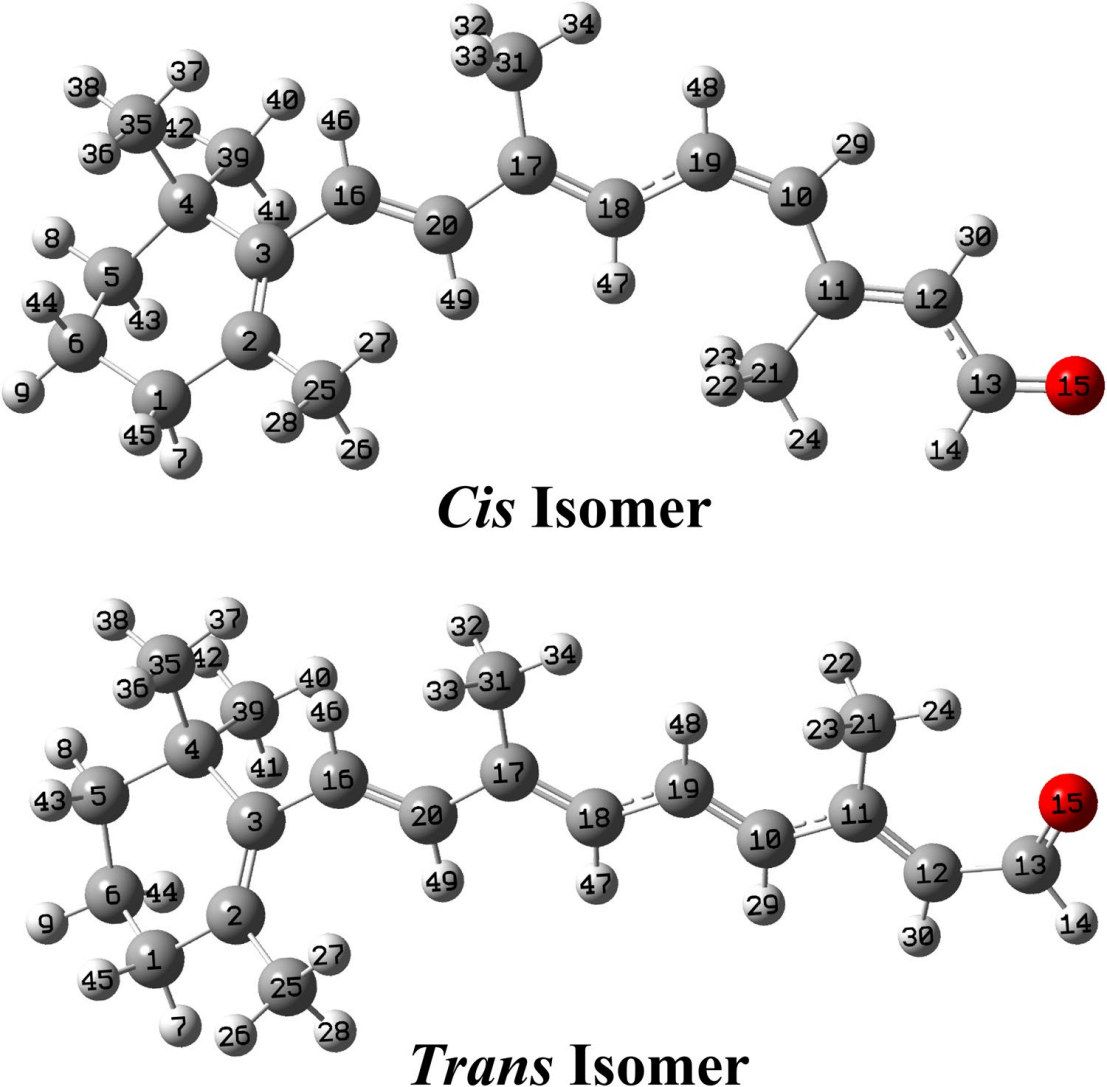
Optimized geometries at B3LYP/6–311 +  + G(d,p) level of theory for 11-*Cis* retinal and *Trans* retinal with their numberings.

The optimized molecular structures of 11-*Cis* retinal and *Trans* retinal are shown in Fig. 4.

Comparing the optimized parameters of both structures, it is observed that the C11 = C12 and C12–C13 bond lengths in the *Cis* form are shorter than in the *Trans* form, while in the 11-*Cis* form, the C19-C10-C11 bond angle is 132.7°, changing to 125.9° in the *Trans* form. Other significant differences between the two forms are observed in the C10-C11-H bond angles, where the values for the *Cis* and *Trans* isomers are 116.7° and 117.8°, respectively. Moreover, the C18-C19-C10-C11 dihedral angles show values of 0.8° and 179.9° in the 11-*Cis* and *Trans* isomers, respectively.

The stability of 11-*Cis* retinal and 9-*Cis* retinal along with their protein part (opsin) were also calculated. The presence of opsin reduces the stability value from 5.7 kcal/mol in the gas phase to 3.8 kcal/mol in water. Considering that 11-*Cis* retinal is present in the structure of rhodopsin^58^, and it converted to *Trans* retinal through a molecular switch, we studied the stabilities using second-order perturbation energies E^(2)^, AIM study, IR, Raman and NMR spectra, and conductivity properties of these two isomers.

### NBO and AIM studies

Possible intra-molecular and hydrogen bond interactions in both isomeric forms examined using NBO and AIM calculations with the NBO and AIM 2000 programs^42,50^. The selected second-order perturbation energies E^(2)^ (donor → acceptor) obtained in the gas phase at B3LYP/6–311 +  + G(d,p) level of theory are presented in Table S1** (**see supplementary materials). These results indicate transitions from various types of bonding (C–C, C–O, and O orbitals) to anti-bonding orbitals such as π → π*, σ → σ*, and LP → σ* transitions. The total energy of interactions shows that the stabilization through resonance in the *Cis* form (690.54 kJ/mol) in the gas phase is greater than in the *Trans* form (683.85 kJ/mol).

Bader’s theory with the AIM 2000 program was used to obtain the electron density, Laplacian values, and the *|λ1|/λ3* ratio at the same level of theory in the bond critical points (BCPs) and ring critical points (RCPs). The interaction is ionic or polar covalent if the ratio *λ1/λ3* < 1 and ∇^2^*ρ(r)* > *0* (closed-shell interaction). The structures of 11-*Cis* and *Trans* isomers in the gas phase within their bond critical points (BCPs) and ring critical points (RCPs) are given in Fig. S3 (see supplementary materials). The RCPN corresponds to the new RCP formed due to new interactions between atoms that create a hypothetical ring (see Fig. S3 in supplementary materials). The *Cis* form exhibits four new interactions: two of the C–H···H type (C19–H48…H34 and C13–H14…H24) and two of the C–H···C type (C18–H47…C21 and C25–H27…C20). In contrast, the *Trans* form shows only three interactions: one of the C–O···H type (C13–O15…H24) and two of the C–H···H type (C19–H48…H34 and C16H46…H37). These additional interactions in the *Cis* form support its higher stability, as suggested by NBO calculations.

### Molecular electrostatic potentials

The different reaction sites in both *Cis* and *Trans* isomers can be predicted using molecular electrostatic potentials (MEP). The diverse red, blue, and green colors observed on the mapped MEP surfaces indicate nucleophilic, electrophilic, and inert regions, respectively, for the two isomers (see Fig. S4 in supplementary materials). The *Cis* isomer shows a higher energy value in the gas phase (± 0.062 a.u.) compared to the *Trans* form (± 0.058 a.u.) in the same medium. These maps allow us to visualize similar charge distributions on the two isomers. The red colorations on the C = O bonds of both forms indicate nucleophilic sites, while blue colors on the H atoms of various C-H and CH_3_ groups represent electrophilic sites. The surfaces with green colors indicate inert sites where no reactions occur.

### Vibrational analysis

**Figure 5:**
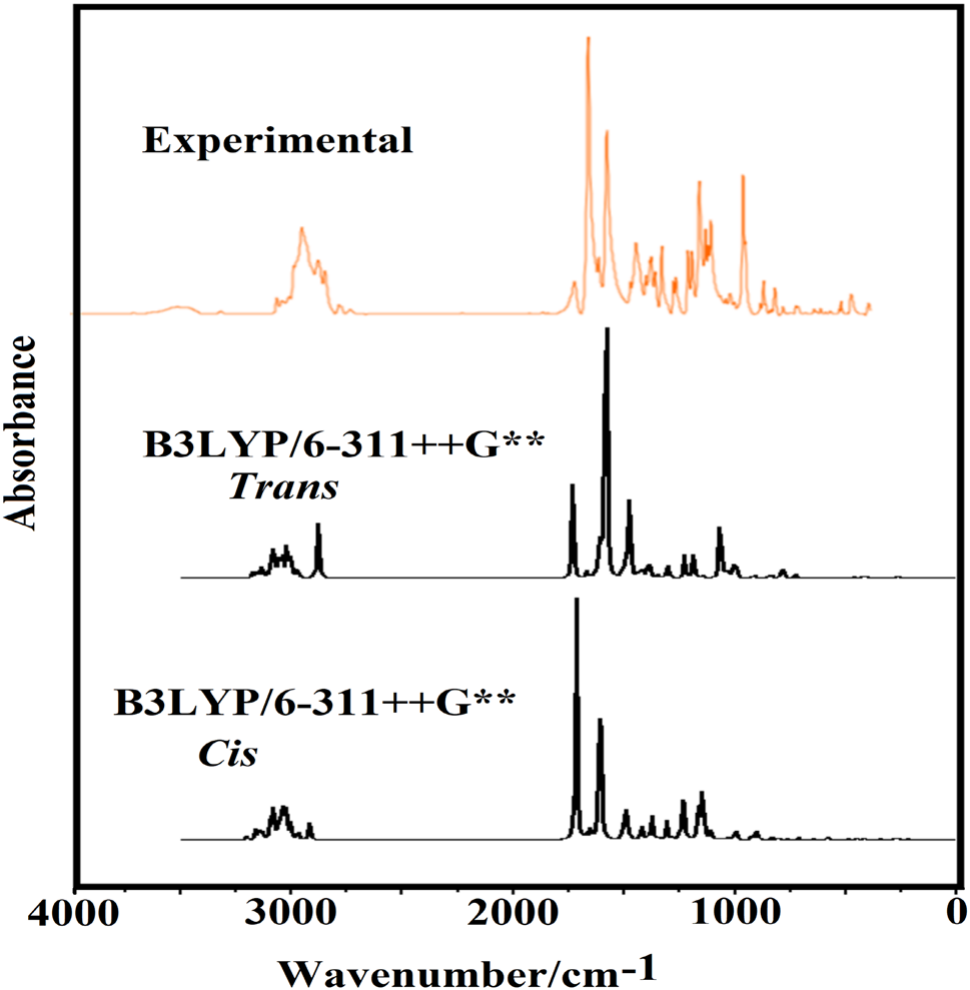
Experimental and calculated IR spectra for 11-*Cis* and *Trans* retinal at B3LYP/6–311 +  + G(d,p) level of theory.

**Figure 6:**
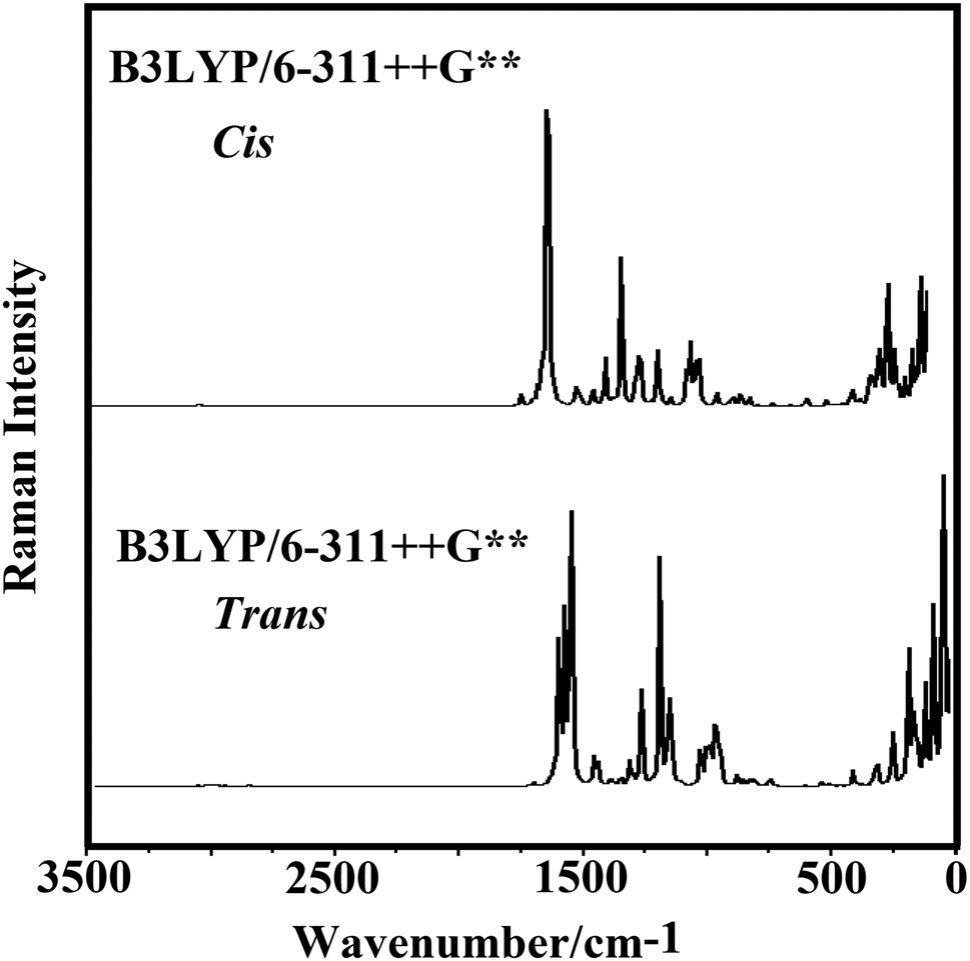
Predicted Raman spectra for 11-*Cis* and *Trans* retinal at B3LYP/6–311 +  + G(d,p) level of theory.

The structures of *Cis* and *Trans* isomers in the gas phase with *C*_*1*_ symmetries were optimized at B3LYP/6–311 +  + G** level of calculations. Due to the presence of 49 atoms in the structures, 141 normal vibration modes are expected. Figure 5 shows comparisons of the experimental infrared spectrum for the *Trans* form in the solid phase taken from Ref.^59^ with the prediction for the two *Cis* and *Trans* isomers in the gas phase at the mentioned level of theory. The best correlation is between the calculated IR spectra for the *Trans* isomer and the experimental spectrum. The predicted Raman spectra for both isomers corrected from activities to intensities are shown in Fig. 6^60^. The vibrational assignments have been performed using the SQMFF methodology and the Molvib program as detailed in the model and computational methods section. PED contributions ≥ 10% have been considered. Table S2 (see supplementary materials) shows the experimental and calculated wavenumbers and their assignments for *Cis* and *Trans* isomers in the gas phase. According to Fig. 5 and Table S2, difference in positions and intensities of predicted bands by SQM calculations for the two isomers and experimental bands are observed in the lower wavenumber region.

#### Assignments

In general, different assignments are predicted for the two isomers, and some vibration modes are observed coupled with other modes. According to SQM calculations, the expected C13-H14 stretching mode of aldehyde for 11-*Cis* retinal is predicted at 2800 cm^–1^, while for the *Trans* form, it isat 2762 cm^–1^. The band at 2763 cm^–1^ can be assigned to those vibration modes, as detailed in Table S2. The strong IR band at 1714 cm^–1^ is assigned to the C13=O15 stretching modes of both forms, while the group of intense IR bands between 1654 and 1572 cm^–1^ are assigned to the five C=C stretching modes predicted for the two forms. The expected antisymmetric and symmetric deformation modes for the CH_2_ and CH_3_ groups are assigned as predicted by SQM calculations between 1449 and 1329 cm^–1^. In both isomers, some rocking modes of CH_3_ groups are predicted by calculations in the 1031–995 cm^–1^ region, while for the *Cis* form, one of these modes is predicted at 1154 cm^–1^.

The twisting modes of CH_2_ and CH_3_ groups for both forms are predicted by calculations in approximately the same regions, while the deformations and torsions of rings are predicted in different regions.

### NMR study

The ^1^H and ^13^C-NMR spectra and chemical shifts of 11-*Cis* and *Trans*-retinal were predicted using the B3LYP/6–311 +  + G(d,p) level of theory in gas phase, and the results are compared with the experimental ones^35^ in CDCl_3_ as solvent in Table S3 (see supplementary materials), using the root-mean-square deviation (RMSD) values. For the H nuclei of both forms (0.88 and 0.86 ppm), promising concordance is observed. The chemical shift values for C10, C12, C21, C35, and C39 atoms in *Cis* and *Trans* forms show a significant difference. The chemical shifts of C10 are 139.8 ppm and 145.8 ppm for *Cis* and *Trans,* respectively. Contrary to this, the values of chemical shifts for C12 and C21 in the *Cis* form are higher than in the *Trans* form, at 139.5 (21.7) ppm and 133.1 (18.5) ppm (values in parentheses correspond to C21), respectively. Additionally, the chemical shifts of H22, H23, and H24 are expected to differ between the *Cis* and *Trans* structures. The average chemical shift of H22, H23, and H24 are 3.26 ppm for 11-*Cis* and 3.46 ppm for the *Trans* form.

### Electronic spectra

**Figure 7:**
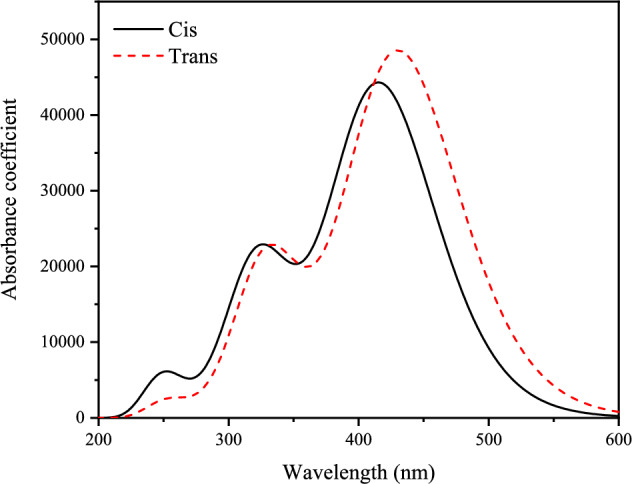
Calculated UV–Vis spectra of 11-*Cis* and *Trans* retinal.

The B3LYP/6–311 +  + G(d,p) level of theory was used to calculate the absorption spectra of 11-*Cis* and *Trans* retinal using the TD-DFT method. Wavelength (λ), oscillator strength (ƒ), and major contributions of the calculated transitions are given in Table S4 (see supplementary materials). The UV–Vis spectrum of 11-*Cis* and *Trans* retinal is shown in Fig. 7.

According to Fig. 7, the values of wavelengths and absorbance coefficients in *Cis* and *Trans* forms are not significantly different. The highest absorption wavelength at 416 and 430 nm for 11-*Cis* and *Trans* isomers is assigned to H → L (95%) contribution for both *Cis* and *Trans*. The second bands at 327 and 333 nm are attributed to H-1 → L (about 86% for both isomers) contribution for both forms. The third band at 254 nm is assigned to H-3 → L(43%) contribution for 11- *Cis* retinal and H-3 → L (58%) and H → L + 1(21%) contributions for *Trans* retinal.

### Conductivity properties

**Figure 8:**
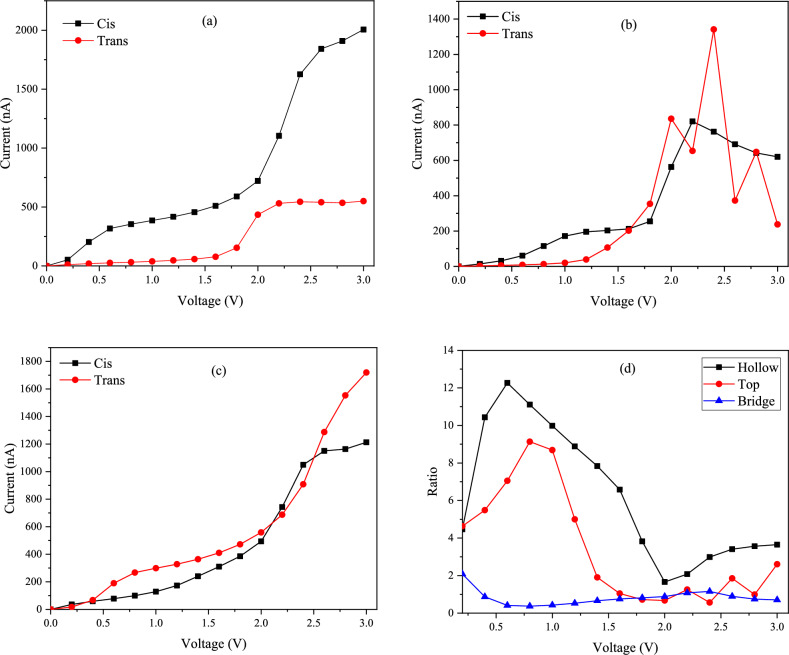
The I-V characteristics of the molecular switches on Ag (111) electrode with (**a**) hollow, (**b**) top, (**c**) bridge sites, and (**d**) current switching ratios as a function of applied bias.

The importance of the mentioned molecular switch between 11-*Cis* and *Trans* in rhodopsin led us to calculate the conductivity properties of 11-*Cis* and *Trans* retinal with different connection sites (hollow, top, bridge) and different electrodes (Ag, Au, Pt). The calculated current–voltage (I-V) curves for both 11-*Cis* and *Trans* forms with different connection sites are shown in Fig. 8. In Fig. 8a, the current–voltage diagram for both 11-*Cis* and *Trans* forms is examined at the hollow site with the Ag electrode. It is evident that the conductivity of the *Cis* form is higher than that of the *Trans* form. In both forms, the current increases with the voltage. The highest slope of the current change occurs between 1.8 V and 2.2 V. In Fig. 8b, the top connection site is investigated. Up to a voltage of about 1.6 V, the current value for the *Cis* form is greater than the *Trans* form. At higher voltages, the current in the *Trans* form increases irregularly. At voltages higher than 2 V, a decrease in the current is observed in both forms. Due to the high fluctuation in conductivity, the top connection site cannot reliably compare the conductivity of the two forms, unlike the hollow site. In Fig. 8c, The current–voltage graph in the bridge site is examined. The trend of the current–voltage diagram in the bridge site is similar to the hollow site, with the difference that up to a voltage of about 2 V, the conductivity in *Trans* retinal is slightly higher than 11- *Cis*. After that and up to a voltage of about 2.5 V, the conductivity in 11-*Cis* becomes higher than *Trans* retinal. To find out which junction has the best performance, the current ratio of the *Cis* to *Trans* form (${\text{I}}_{\text{cis}}/{\text{I}}_{\text{trans}}$) was calculated, as shown in Fig. 8d. According to the obtained ratio values, the hollow site has the highest ratio. The highest ratio of *Cis* to *Trans* current is observed in the hollow connection site at 0.6 V, which is about 12.3.

**Figure 9:**
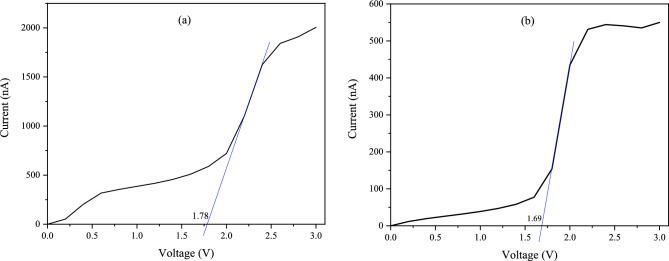
The trendline of the current–voltage diagrams and the intersection of the extrapolation with the x-axis in the Ag electrode with the hollow connection site: (**a**) 11- *Cis* retinal, (**b**) *Trans* retinal.

The hollow site exhibited the best performance, so we used this connection site in subsequent calculations with Au and Pt electrodes. To better distinguish the conductivity of the *Cis* form from the *Trans* form, we used the trendline technique. As seen in Fig. 9, our current–voltage graphs for the desired molecules were S-shaped. This unique feature allowed us to draw the trendline for both *Cis* and *Trans* forms in the silver electrode on the hollow site, and by extrapolating it, we found the difference between the two forms. As shown in Fig. 9a, in 11- *Cis* retinal, the x-axis trendline intersects at 1.78 V, but this value for the *Trans* retinal is 1.69 V, as shown in Fig. 9b. This confirms that the electrical conductivity in the *Cis* form is higher than the *Trans* form.

**Figure 10:**
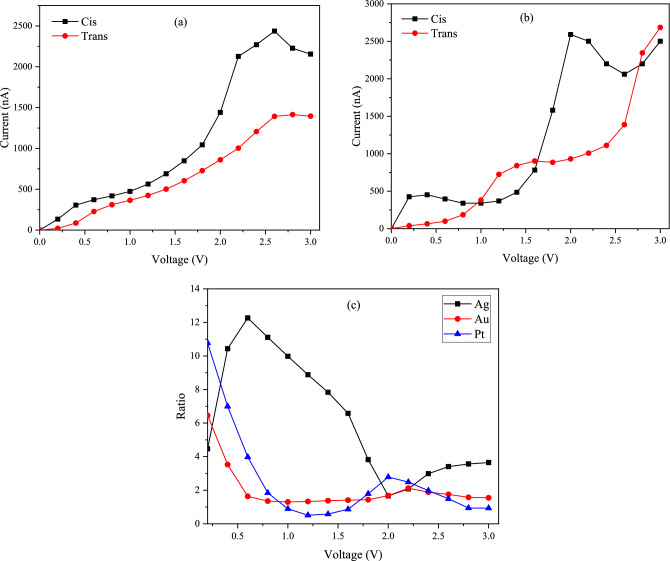
The calculated I–V characteristics of the molecular switches with the hollow site: (**a**) Au, (**b**) Pt, and (**c**) current ratios of the molecular switches with different electrodes.

In Fig. 10, we examined the 11-*Cis* and *Trans* retinal current–voltage plots at the hollow junction in different electrodes. Figure 10a shows the current–voltage diagram in the gold electrode and hollow position. The amount of current for the *Cis* form at all voltages is higher than the *Trans* form. In Fig. 10b, the Pt electrode is investigated. In this electrode, up to a voltage of about 1 V, the conductivity of the *Cis* form is significantly higher than the *Trans* form. but at voltages between 1 and 1.6 V, the current in the *Trans* form is higher than the *Cis* form. At voltages higher than 1.6 V to about 2.7 V, the conductivity of the *Cis* form is higher than the *Trans* form again. Typically, conduction diagrams are low at first and then increase as an exponential function^9,10^. However, as seen in Fig. 10b, we observe different behavior in the Pt electrode due to high fluctuation, and this electrode, unlike the Ag and Au electrodes, does not show the difference in conductivity between the two forms well.

We also calculated the *Cis* to *Trans* current ratio in three electrodes (Ag, Au, Pt) to check the performance of different electrodes, as shown in Fig. 10c. According to the obtained results, the silver electrode has the highest ratio at almost all voltages. It should be noted that the highest current measured in our calculations occurred in the Pt electrode at the hollow position for the *Trans* form, reaching approximately 2690 nA.

**Figure 11:**
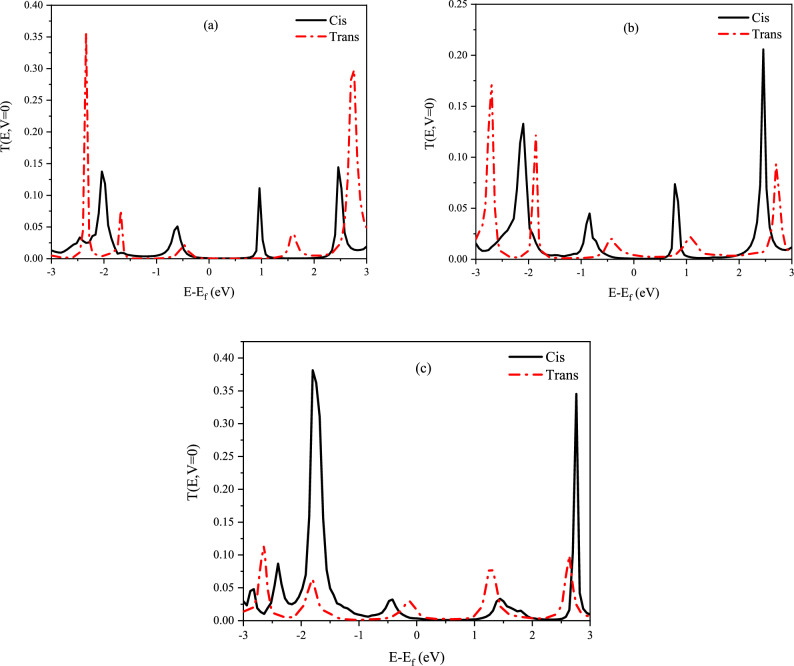
The transmission spectra of the molecular switch at zero bias with a hollow site on (**a**) Ag, (**b**) Au, and (**c**) Pt electrode.

The difference in conductivity of 11-*Cis* and *Trans* retinal can be explained by the transmission diagrams and the gap between the highest occupied molecular orbitals (HOMO) and the lowest unoccupied molecular orbitals (LUMO). The transmission spectra were calculated for 11-*Cis* and *Trans* retinal on three electrodes with hollow sites (see Fig. 11). In the calculations, the average Fermi level is set to zero, and according to the Landauer – Büttiker formula, only electrons with energy around the Fermi level play a significant role in the current transfer. According to the Fig. 11a,b, which relate to the Ag and Au electrodes, the transmission coefficients and the area under the peak in electrons close to the Fermi level 11-*Cis* are much higher than the *Trans* form, confirming the higher conductivity in the *Cis* form. In Fig. 11c, the transmission spectra of the Pt electrode are also given. This figure confirms that the Pt electrode does not show the difference in conductivity between the two forms as well as Ag and Au electrodes.

**Figure 12:**
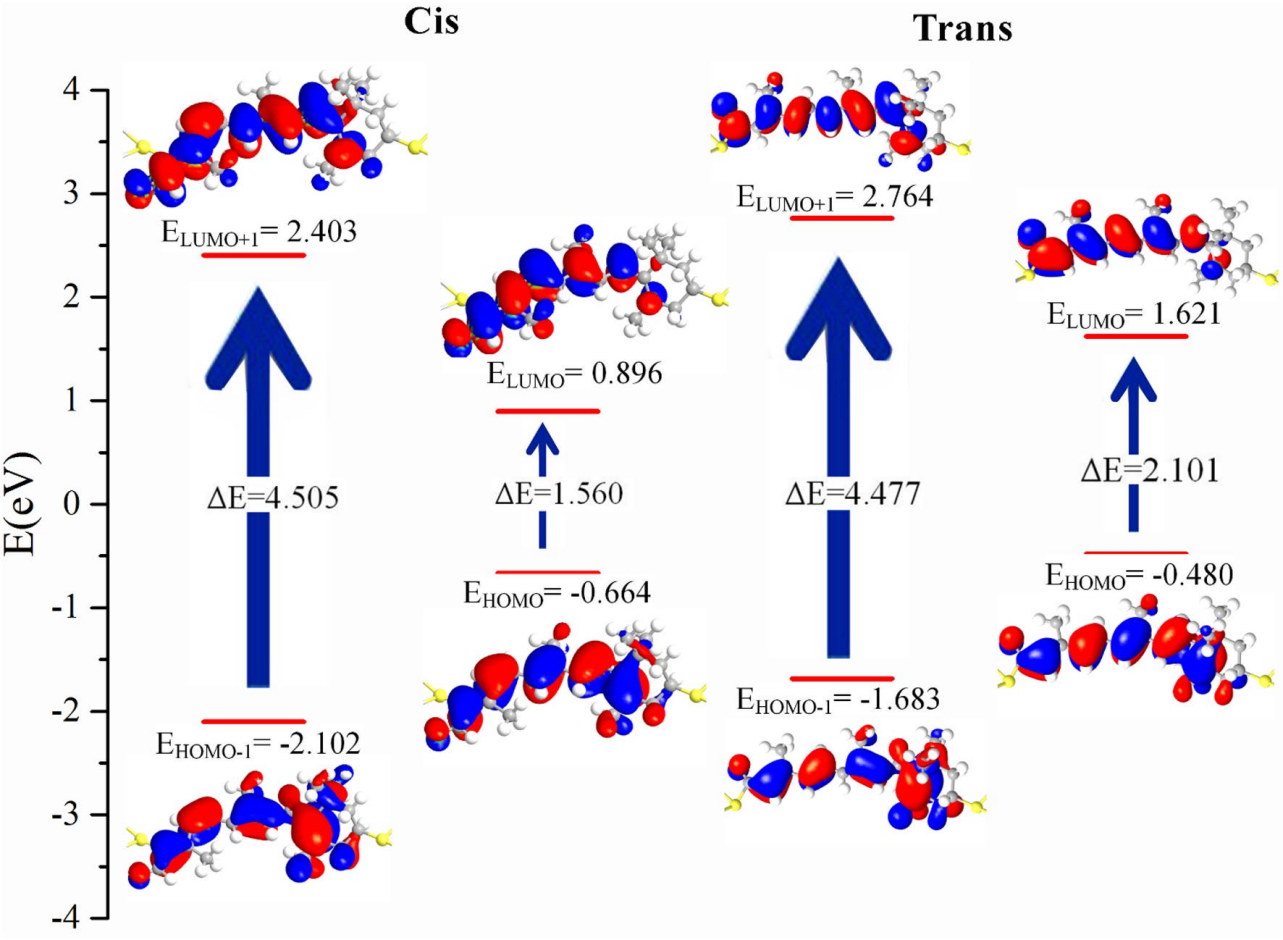
The spatial distribution of the MPSH states corresponding to HOMO, LUMO, HOMO-1, and LUMO + 1 of 11-*Cis* and *Trans* retinal on Ag electrode with hollow site.

The molecular projected self-consistent Hamiltonian (MPSH) was used to investigate conduction channels. The spatial distribution of the MPSH states and the energy values of the frontier molecular orbitals (HOMO-1, HOMO, LUMO, and LUMO + 1) of 11-*Cis* and *Trans* retinal on Ag electrode with the hollow site are shown in Fig. 12. The energy levels of 11-*Cis* are -0.664 and 0.896 eV for HOMO and LUMO, respectively. These values for the *Trans* form are − 0.480 and 1.621 eV. The HOMO–LUMO gap in the *Cis* form is 1.560 eV, and in the *Trans* form is 2.101 eV, confirming the results obtained from the voltage-current diagrams. We know that the smaller the HOMO–LUMO gap distance, the more electron transfer occurs, where the HOMO–LUMO orbital distance in the *Cis* form is smaller than in the *Trans* form, and therefore the *Cis* form is more conductive. According to Fig. 12, the HOMO and LUMO orbitals in the *Cis* form are more delocalized than the *Trans* isomer, providing an additional pathway for electron transport, confirming the higher conductivity in the *Cis* form. These frontier orbitals (HOMO and LUMO) provide the main electronic transfer channel for the *Cis* form and involve little barrier to electron transfer.

## Conclusion

In this research, the structures and theoretical spectroscopic properties of 11-*Cis* and *Trans* retinal isomers have been studied using the B3LYP/6–311 +  + G** calculations. Complete vibrational assignments for both forms are reported using the scaled quantum mechanical force field (SQMFF) methodology. In addition, the conductivity properties of both isomers, constituents of rhodopsin, were studied by using the DFT, NEGF, and TD-DFT. I-V curves, transmission spectra, and spatial distribution of MPSH showed that the conductivity of the *Cis* form is higher than that of the *Trans* form. The unique feature of the studied molecules was that the I-V graphs were S-shaped, rarely seen in other works. The I-V results were confirmed by extrapolating the trendlines of the current–voltage plots. The summation of second-order interaction energies (E^2^) in 11-*Cis* is more than that in *Trans,* confirming more electron delocalization in *Cis*. Higher electron delocalization causes more conductivity in the *Cis* isomer. Among the three electrodes and three types of connection sites examined, the silver electrode and the hollow connection site had the highest *Cis* to *Trans* current ratio. The highest *Cis* to *Trans* current ratio observed in the silver electrode and the hollow connection site was about 12.3 at 0.6 V. Therefore, the title molecules can be considered as molecular switches.

### Supplementary Information

Supplementary Information.

## Data Availability

All data generated or analyzed during this study are included in this published article (and its Supplementary Information files).
