# Supplementary material for: Theoretical study on the structure, spectroscopic, and current–voltage behavior of 11-Cis and Trans retinal isomers in rhodopsin
Source: Sci Rep. 2024 May 30;14:12452. doi: 10.1038/s41598-024-63249-8 (PMC11140004; doi:10.1038/s41598-024-63249-8)
Supplement: Supplementary file 1 — Supplementary Information. [file 41598_2024_63249_MOESM1_ESM.pdf]

MATERIAL SUPORTING TO

Theoretical investigation on the structure, spectroscopic, and current-voltage behavior of 11-*Cis* and *Trans* retinal isomers in Rhodopsin as a molecular switch

Amin Hamedian [a], Mohammad Vakili [a*], Silvia A. Brandán [b], Mahmood Akbari [c,], Ayoub Kanaani [d], Vahidreza Darugar [a]

[a] *Department of Chemistry, Faculty of Science, Ferdowsi University of Mashhad, Mashhad 91775-1436, Iran*
[b] *Cátedra de Química General, Instituto de Química ,Inorgánica Facultad de Bioquímica, Química y Farmacia, Universidad Nacional de Tucumán, Ayacucho 471, 4000, Tucumán, Argentina.*
[c] *UNESCO-UNISA-ITL Africa Chair in Nanoscience and Nanotechnology (U2ACN2), College of Graduate Studies, University of South Africa (UNISA), Pretoria, South Africa*
[d] *School of Chemistry, Damghan University, Damghan 36716-41167, Iran*

Corresponding author: *vakili-m@um.ac.ir*

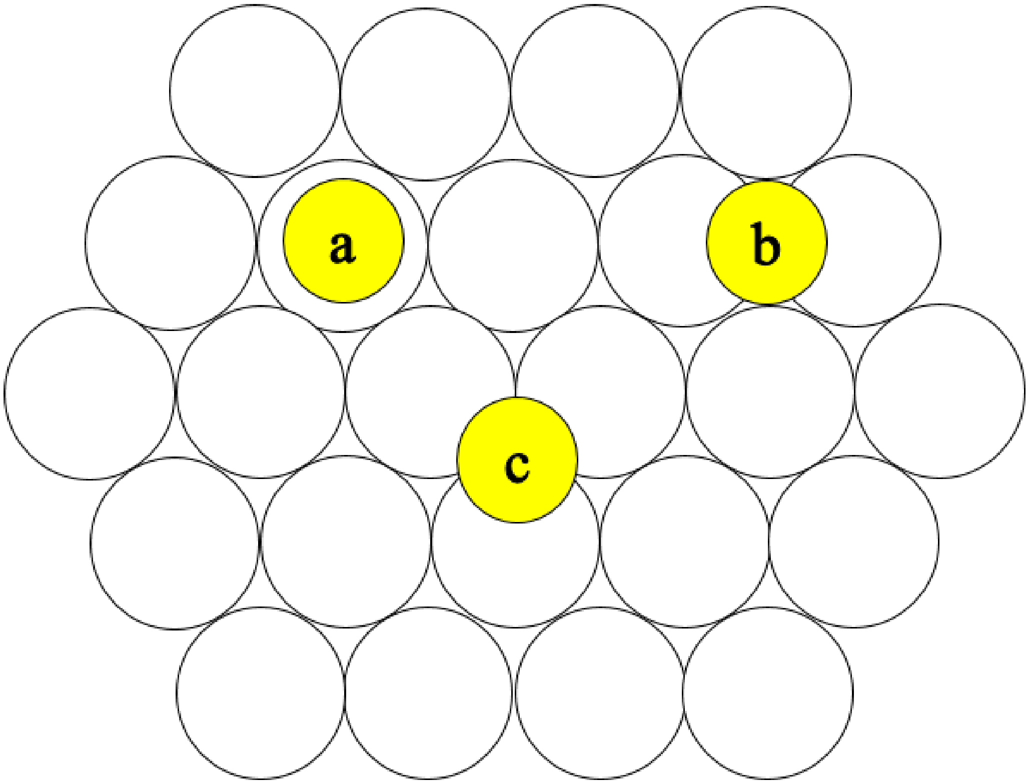

Figure S1. Scheme of three types of connection sites: **a)** top, **b)** bridge, **c)** hollow

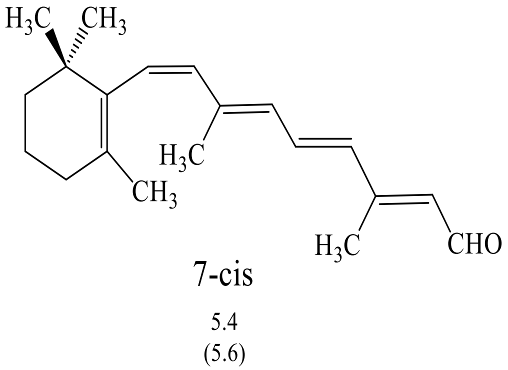

7-cis

5.4
(5.6)

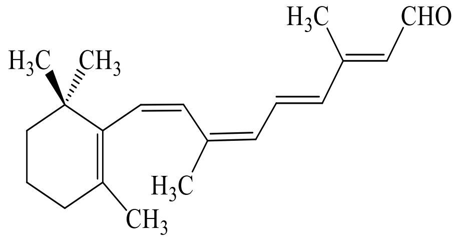

7,9-dicis

6.1
(6.6)

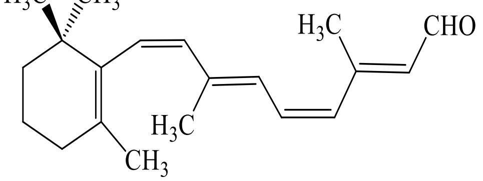

7,11-dicis

11.5
(11.9)

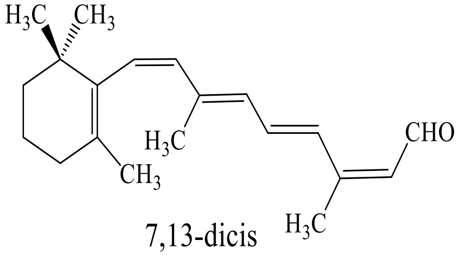

7,13-dicis

5.9
(6.2)

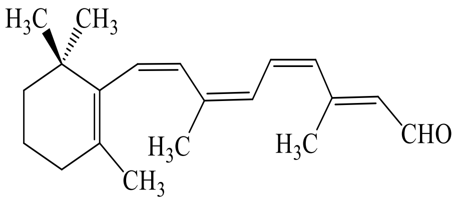

7,9,11-tricis

12.1
(12.7)

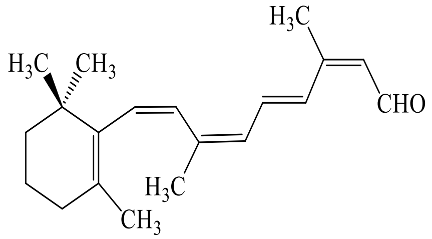

7,9,13-tricis

6.6
(7.2)

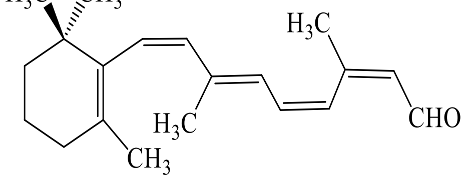

7,11,13-tricis

9.0
(9.9)

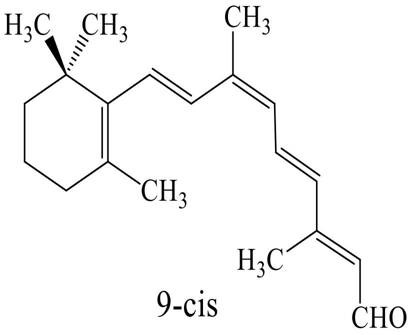

9-cis

0.5
(0.6)

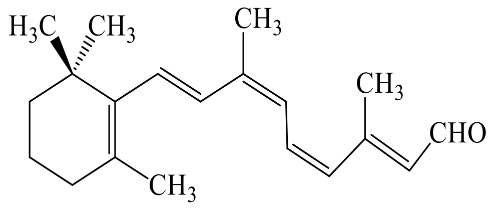

9,11-dicis

6.1
(6.5)

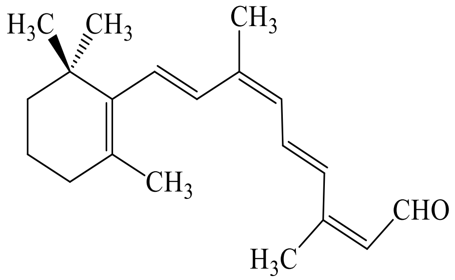

9,13-dicis

1.0
(1.2)

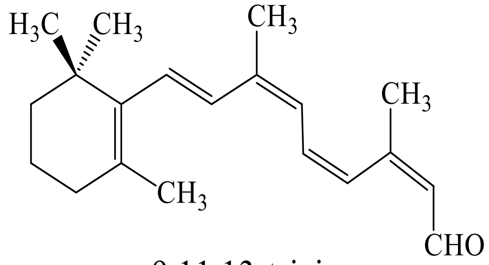

9,11,13-tricis

3.4
(4.3)

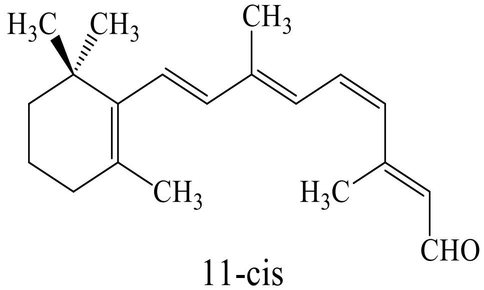

11-cis

6.1
(6.3)

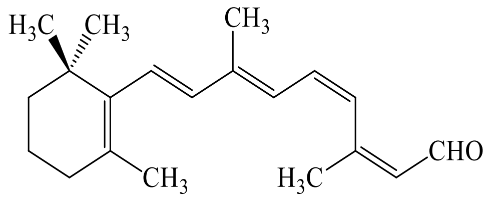

11,13-dicis

3.5
(4.3)

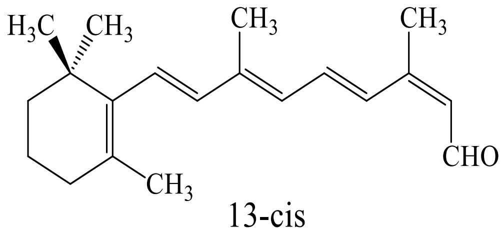

13-cis

0.4
(0.7)

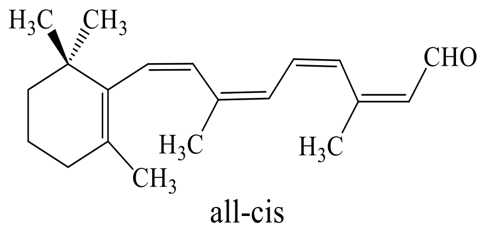

all-cis

9.7
(10.9)

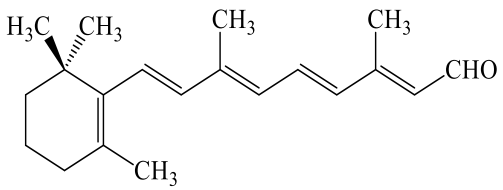

Trans

0.0
(0.0)

Figure S2. Structures of all 15 *Cis* isomers and one *Trans* isomer with their relative energies in gas phase and water, as the solvent, in kcal/mol. (The values in parentheses are the relative energies in water).

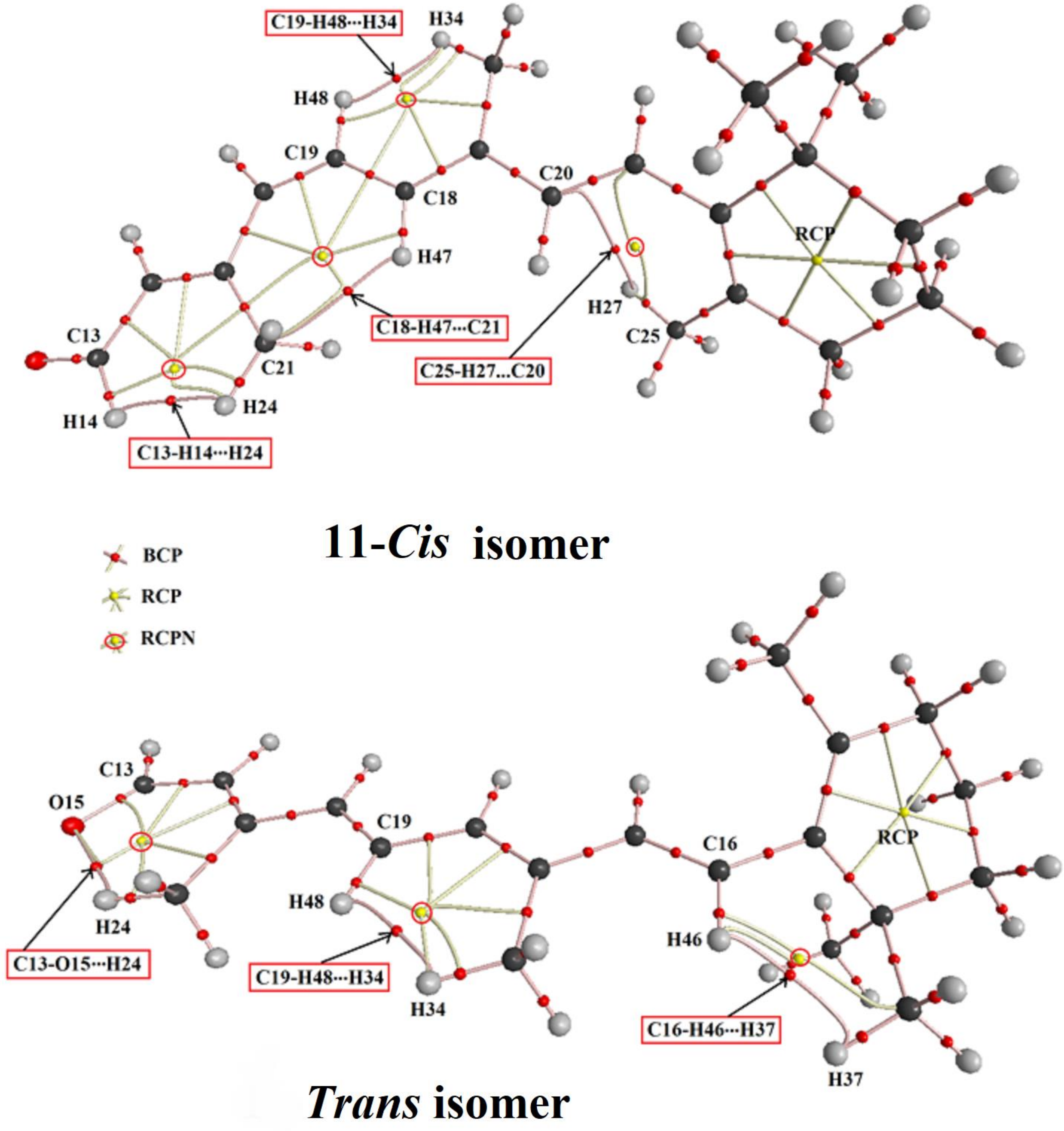

Figure S3. Details of the molecular models for 11-*Cis* and *Trans* isomers in the gas phase using the B3LYP/6-311++G** method showing the geometries of all their bond critical points (BCPs) and ring critical points (RCPs).

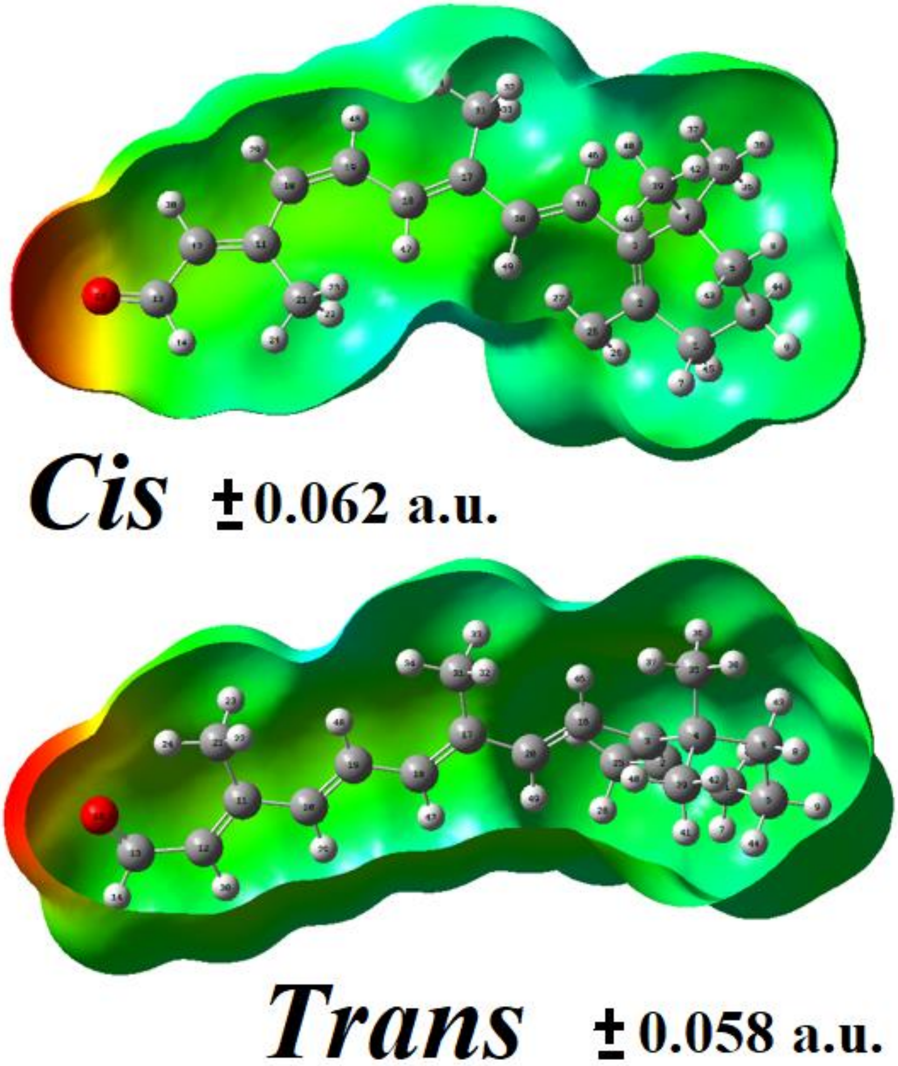

Figure S4. Calculated electrostatic potential surfaces on the molecular surface of 11-*Cis* and *Trans* isomers in the gas phase by using the B3LYP/6-311++G** method. Isodensity value of 0.005.

Table S1. The selected second-order perturbation energies $E^{(2)}$ (donor$\rightarrow$acceptor) obtained for *Cis* and *Trans* isomers in the gas phase at B3LYP/6-311++G(d,p) level of theory.

Donor	Type	Acceptor	Type	Cis	Trans
C_1-C_2	σ	C_3-C_{16}	σ^*	22.57	21.74
C_2-C_3	π	C_{16}-C_{20}	π^*	35.53	35.11
C_{17}-C_{18}	π	C_{16}-C_{20}	π^*	64.79	65.63
C_{11}-C_{12}	π	C_{10}-C_{19}	π^*	68.13	63.54
C_{11}-C_{12}	π	C_{13}-O	π^*	91.96	97.81
C_{13}-O	π	C_{11}-C_{12}	π^*	26.33	20.48
C_{16}-C_{20}	π	C_2-C_3	π^*	30.93	33.02
C_{16}-C_{20}	π	C_{17}-C_{18}	π^*	67.30	66.88
C_{17}-C_{18}	π	C_{10}-C_{19}	π^*	80.67	73.99
C_{17}-C_{18}	π	C_{16}-C_{20}	π^*	64.79	65.63
O	LP(1)	C_{13}	$RY^*(1)$	54.34	56.85
O	LP(2)	C_{13}	$RY^*(2)$	14.63	14.21
O	LP(2)	C_{12}-C_{13}	σ^*	68.55	68.97
**Total Energy**				**690.54**	**683.85**

*Energy in kJ/mol.

Table S2. Observed and calculated wavenumbers (cm^{-1}) and assignments for *Cis* and *Trans* isomers in the gas phase using the hybrid B3LYP/6-311++G** Method.

Experimental [b]	B3LYP/6-311++G** Method			
	CIS		*TRANS*	
TRANS	SQM	Assignments [a]	SQM [c]	Assignments [a]
3468w	3073	νC18-H47	3048	νC19-H48
3288w	3032	νC19-H48	3036	$\nu_a CH_3$(C21)
3037w	3024	νC12-H30	3028	νC16-H46
3018w	3017	νC20-H49	3014	νC20-H49
	3014	$\nu_a CH_3$(C21)	3010	νC12-H30
	3010	νC10-H29	3004	$\nu_a CH_3$(C31)
	3007	νC16-H46	3001	νC10-H29
	3002	$\nu_a CH_3$(C31)	2998	$\nu_a CH_3$(C25)
2987w	3001	$\nu_a CH_3$(C25)	2995	νC18-H47
	2970	$\nu_a CH_3$(C35)	2968	$\nu_a CH_3$(C35)
	2969	$\nu_a CH_3$(C39)	2965	$\nu_a CH_3$(C39)
	2959	$\nu_a CH_3$(C21)	2957	$\nu_a CH_3$(C39)
	2957	$\nu_a CH_3$(C39)	2953	$\nu_a CH_3$(C35)
	2953	$\nu_a CH_3$(C35)	2943	$\nu_a CH_3$(C31)
	2944	$\nu_a CH_3$(C31)	2943	$\nu_a CH_3$(C21)
2927s	2935	$\nu_a CH_2$(C6)	2932	$\nu_a CH_2$(C6)
	2921	$\nu_a CH_3$(C25)	2921	$\nu_a CH_2$(C5)
	2920	$\nu_a CH_2$(C5)	2917	$\nu_a CH_3$(C25)
	2911	$\nu_s CH_3$(C21)	2902	$\nu_s CH_3$(C31)
	2907	$\nu_a CH_2$(C1)	2901	$\nu_s CH_2$(C6)
	2903	$\nu_s CH_3$(C31)	2900	$\nu_s CH_3$(C21)
	2901	$\nu_s CH_3$(C35)	2899	$\nu_s CH_3$(C35)
	2897	$\nu_s CH_2$(C6)	2893	$\nu_s CH_3$(C39)
	2895	$\nu_s CH_3$(C39)	2887	$\nu_a CH_2$(C1)
	2881	$\nu_s CH_2$(C5)	2881	$\nu_s CH_3$(C25)
2857m	2881	$\nu_s CH_3$(C25)	2879	$\nu_s CH_2$(C5)
2826m	2851	$\nu_s CH_2$(C1)	2854	$\nu_s CH_2$(C1)
2763w	2800	νC13-H14	2762	νC13-H14
1714m	1652	νC13-O15	1670	νC13-O15
1654vs	1612	νC2-C3,νC16-C20	1611	νC2-C3,νC16-C20
1607m	1592	νC2-C3,νC16-C20	1595	νC2-C3,νC19-C10
1572s	1576	νC11-C12	1576	νC17-C18
1572s	1555	νC19-C10	1553	νC19-C10
1572s	1527	νC19-C10νC17-C18	1527	νC11-C12
1462sh	1448	$\delta_a CH_3$(C35)	1449	$\delta_a CH_3$(C35), $\delta_a CH_3$(C39)
1440m	1440	$\delta_a CH_3$(C35),	1444	$\delta_a CH_3$(C35), $\delta_a CH_3$(C39)
	1438	βC10-H29	1440	$\delta_a CH_3$(C21)
	1435	δCH_2(C6)	1435	δCH_2(C6)
	1432	$\delta_a CH_3$(C39)	1433	δCH_2(C5)

	1431	δ_aCH_3(C25)	1430	δ_aCH_3(C31)
	1431	δ_aCH_3(C21)	1428	δ_aCH_3(C25)
1427sh	1426	δ_aCH_3(C21)	1423	δ_aCH_3(C35),δ_aCH_3(C25)
	1425	δ_aCH_3(C25)	1423	δ_aCH_3(C25)
	1423	δ_aCH_3(C39),	1420	δ_aCH_3(C31)
	1423	δ_aCH_3(C31)	1417	δCH_2(C5), δ_aCH_3(C39)
1419sh	1422	δ_aCH_3(C31)	1415	δ_aCH_3(C21)
	1417	δCH_2(C5)	1406	δCH_2(C1)
	1408	δCH_2(C1)	1404	βC13-H14
1397m	1390	βC13-H14	1380	βC18-H47,νC17-C31
1376m	1376	δ_sCH_3(C31),βC18-H47	1366	βC12-H30, δ_sCH_3(C31)
	1367	δ_sCH_3(C21), νC11-C21	1363	wagCH_2(C5),wagCH_2(C1)
	1366	wagCH_2(C5),wagCH_2(C	1358	wagCH_2(C1)
1356m	1361	wagCH_2(C1)	1349	wagCH_2(C6)
	1349	wagCH_2(C6)	1347	δ_sCH_3(C25)
	1349	δ_sCH_3(C25)	1344	δ_sCH_3(C31)
	1342	wagCH_2(C5)	1338	wagCH_2(C5)
	1338	δ_sCH_3(C31)	1333	δ_sCH_3(C21)
	1336	δ_sCH_3(C39),	1332	δ_sCH_3(C39),δ_sCH_3(C35)
1323m	1329	δ_sCH_3(C21)	1314	βC16-H46,νC16-C20
1313sh	1309	βC16-H46,βC20-H49	1307	βC16-H46,βC10-H29
	1294	βC16-H46,βC20-H49	1296	βC20-H49
1274m	1271	βC19-H48	1270	ρCH_2(C5),ρCH_2(C1)
1264m	1268	ρCH_2(C5),ρCH_2(C1)	1265	βC19-H48
	1250	ρCH_2(C6), βC16-H46	1250	ρCH_2(C6), wagCC_2(C4)
1212s	1214	βC18-H47	1223	νC10-C11,βC12-H30
	1199	ρCH_2(C1)	1196	ρCH_2(C1)
1193s	1198	βC12-H30	1192	βC18-H47,νC17-C20
	1184	ρCH_2(C6)	1180	ρCH_2(C6)
	1166	νC1-C2,νC2-C25	1166	νC1-C2,νC2-C25
1159s	1154	$\rho' CH_3$(C35),ρCH_2(C5)	1161	βR_1(A1)
1133s	1128	νC3-C16	1148	νC18-C19
1110s	1110	νC12-C13	1116	νC3-C16
1097sh	1092	νC4-C35,νC4-C39	1086	νC4-C35,τR_1(A1)
1065sh	1075	νC18-C19	1043	νC5-C6,νC1-C6
1041w	1045	νC5-C6,νC1-C6	1035	ρCH_3(C21)
	1030	$\rho' CH_3$(C21)	1031	$\rho' CH_3$(C21)
	1028	$\rho' CH_3$(C25)	1028	$\rho' CH_3$(C25)
1022w	1025	$\rho' CH_3$(C31)	1024	$\rho' CH_3$(C31)
	1015	γC19-H48,γC10-H29	1017	γC13-H14
	1013	ρCH_3(C31)	1010	ρCH_3(C31)
	1002	γC16-H46,γC20-H49	1005	γC16-H46
1003w	1000	ρCH_3(C35), $\rho' CH_3$(C35)	995	$\rho' CH_3$(C35), ρCH_3(C39)
	991	γC13-H14	991	γC20-H49
	988	γC20-H49	981	γC10-H29,γC19-H48

965s	977	ρCH$_3$(C21)	977	νC12-C13
953sh	963	νC5-C6, ρCH$_3$(C35)	960	ρCH$_3$(C25)
	937	ρCH$_3$(C39),τ$_w$CH$_2$(C6),	940	τ$_w$CH$_2$(C6),ρCH$_3$(C35)
	917	γC18-H47	918	γC18-H47
	912	νC4-C39 ρ'CH$_3$(C35) ρCH$_3$(C35)	910	νC1-C6,γC18-H47
	901	νC4-C35 ρ'CH$_3$(C39) ρCH$_3$(C25)	902	νC4-C35,νC4-C39, ρ'CH$_3$(C39)
887w	899	γC12-H30	894	γC10-H29,γC19-H48
872m	873	γC16-H46	875	γC16-H46,γC20-H49
862sh	862	νC17-C31	860	γC16-H46
838sh	850	νC5-C6,νC4-C35	846	νC5-C6,νC4-C39,νC1-C6
825m	834	δC18C19C10	832	δC11C12C13,νC11-C21 δC12C13O15
	806	νC11-C21,νC10-C11	826	νC17-C31
788w	796	γC10-H29,γC19-H48	822	γC12-H30
	791	τ$_w$CH$_2$(C1), τ$_w$CH$_2$(C5)	791	τ$_w$CH$_2$(C1)
753w	762	τ$_w$CH$_2$(C5),τ$_w$CH$_2$(C1)	764	τ$_w$CH$_2$(C5),νC4-C5
727w	738	δC19C10C11	761	νC11-C21, δC12C13O1
689w	688	δC19C10C11,γC3-C16	699	τ$_w$CH$_2$(C6) νC1-C2
649w	621	γC3-C16	642	γC3-C16
621w528w	578	δC17C18C19	593	δC17C18C19, δC19C10C11 βC17-C31
598w	556	βR$_1$(A1), νC3-C4	556	βR$_1$(A1),νC3-C4
578w	541	τC10-C19,γC11-C21	551	βC17-C31
	524	γC17-C31	530	γC11-C21
	498	βR$_3$(A1)	520	γC17-C31
528w	487	γC17-C31,	479	γC17-C31,βR$_2$(A1)
	476	δC12C13O15	470	βR$_3$(A1)
485w	466	βC17-C31,βR$_3$(A1)	451	βC11-C21,βR$_2$(A1),
436w	443	βR$_2$(A1),δC20C17C18	421	δC10C11C12, δC18C19C10
410sh	424	βC2-C25, wagCC$_2$(C4)	413	wagCC$_2$(C4)
406w	402	γC2-C25	394	γC2-C25
	371	τ$_w$CC$_2$(C4)	366	βC2-C25
	361	τ$_w$C18-C17	358	τ$_w$CC$_2$(C4),ρCC$_2$(C4)
	342	βC3-C16	349	δCC$_2$(C4), βC11-C21
	337	δCC$_2$(C4), ρCC$_2$(C4)	324	τ$_w$C18-C17
	323	βC11-C21,	308	δCC$_2$(C4)
	308	δCC$_2$(C4), ρCC$_2$(C4)	285	δCC$_2$(C4)
	265	δC17C18C19	265	δC11C12C13,τ$_w$CH$_3$(C35)
	260	τ$_w$C20-C17,τ$_w$C18-C17	255	τ$_w$CH$_3$(C35), βC17-C31
	254	τR$_3$(A1)	245	τ$_w$C12-C11,γC12-H30
	251	τR$_3$(A1), τ$_w$C18-C17	244	τ$_w$C12-C11,γC12-H30
	233	τ$_w$CH$_3$(C39)	241	τ$_w$CH$_3$(C35)
	222	τ$_w$CH$_3$(C21)	221	τ$_w$CH$_3$(C25)
	203	τ$_w$CH$_3$(C35), τ$_w$C12-	208	τ$_w$CH$_3$(C39)
	196	τ$_w$CH$_3$(C35)	181	γC3-C16

174	τ_wC12-C11	176	τC19-C18,τ_wC20-C17
166	$\tau_w CH_3$(C25)	160	δC16C20C17
164	δC10C11C12	139	τC10-C19
136	$\tau_w CH_3$(C31)	128	τR_3(A1),τR_1(A1)
128	τC13-C12	116	$\tau_w CH_3$(C31)
118	τR_3(A1),τR_1(A1)	101	τR_2(A1), γC3-C1
107	τR_2(A1)	94	τ_wC12-C11
97	τ_wC16-C3,$\tau_w CH_3$(C25)	74	τC20-C16
68	τC20-C16	62	$\tau_w CH_3$(C21)τC13-C12
56	τR_2(A1), τR_3(A1)	50	τR_2(A1)
40	τC20-C16,τ_wC10-C11	37	τ_wC10-C11
32	δC3C16C20,	27	δC3C16C20
21	τ_wC10-C11	19	τR_2(A1), τR_3(A1)
14	τ_wC16-C3,τC19-C18<br>τ_wC20-C17	13	τ_wC16-C3,τ_wC20-C17

Abbreviations: ν, stretching; β, deformation in the plane; γ, deformation out of the plane; wag, wagging; τ , torsion; ρ, rocking; τw, twisting; δ, deformation; a, antisymmetric; s, symmetric; [a] This work, [b] From Ref [60], [c] From scaled quantum mechanics force field with B3LYP/6-311++G** method.

Table S3. Calculated and experimental ^{1}H and ^{13}C chemical shifts for both *Cis* and *Trans* forms.

	Cis			***Trans***		
Atoms	Calc	Average	Exp [a]	Calc	Average	Exp [a]
H7	3.20			2.99		
H8	2.22			2.22		
H9	2.31			2.36		
H14	11.11		10.08	10.69		10.11
H22	2.99			5.24		
H23	2.94	3.26		2.56	3.46	
H24	3.86			2.57		
H26	2.57			2.65		
H27	3.11	2.60	1.99	3.09	2.67	2.03
H28	2.12			2.28		
H29	6.98		5.92	7.22		6.37
H30	6.59		6.07	6.85		5.98
H32	2.59			2.72		
H33	2.48	2.77	2.35	2.69	2.87	2.33
H34	3.24			3.19		
H36	2.02			1.72		
H37	2.25	1.92	1.72	2.35	1.95	1.72
H38	1.50			1.79		
H40	1.89			1.79		
H41	1.46	1.63	1.03	1.85	1.65	1.04
H42	1.55			1.32		
H43	2.29			2.33		
H44	2.58			2.36		
H45	2.76			2.95		
H46	7.55		6.32	7.64		6.36
H47	7.74		6.54	7.24		6.19
H48	7.83		6.69	8.38		7.14
H49	7.09		6.14	7.20		6.18
**RMSD**	**0.88**			**0.86**		
C1	41.2			41.7		
C2	146.8			149.1		
C3	151.4			152.4		
C4	44.4			45.3		
C5	46.8			46.8		
C6	27.1			26.3		
C10	139.8			145.8		
C11	170.7			170.1		
C12	139.5			133.1		
C13	198.6			201.0		
C16	145.6			144.6		
C17	158.1			157.2		
C18	136.9			140.0		
C19	145.2			147.6		
C20	148.3			150.6		
C21	21.7			18.5		
C25	27.7			27.1		
C31	15.8			17.7		
C35	31.7			34.8		
C39	35.9			32.5		

All chemical shifts are relative to TMS, which its ^{13}C and proton magnetic shielding are 186.943 and 32.648 ppm, respectively; [a] From Ref [36].

Table S4. The calculated wavelength (λ), oscillator strengths (f), and major contributions for both *Cis* and *Trans* forms at B3LYP/6-311++G(d,p) level of theory.

Cis				Trans			
λ_{cal}	f	Major contribution>15%	λ_{exp} [a]	λ_{cal}	f	Major contribution>15%	λ_{exp} [a]
416	1.0855	H→L(94%)	365	430	1.1923	H→ L(95%)	368
327	0.5036	H-1→ L(86%)		333	0.5188	H-1→ L(88%)	
297	0.1104	H→ L+1(68%), H-3→ L(28%)		300	0.0884	H→ L+1(69%)	
254	0.0779	H-3→ L(43%)		259	0.0423	H-3→ L(58%), H→ L+1(21%)	

H-n means n levels lower than HOMO, and L+n means n levels upper than LUMO; [a] From Ref [36].
